# Walk with Me: a protocol for a pilot RCT of a peer-led walking programme to increase physical activity in inactive older adults

**DOI:** 10.1186/s40814-018-0308-2

**Published:** 2018-06-21

**Authors:** Mark A. Tully, Conor Cunningham, Margaret E. Cupples, Duane Farrell, Wendy Hardeman, Ruth F. Hunter, Bob Laventure, Suzanne M. McDonough, Joanne Morgan, Marie H. Murphy, Ellen E. A. Simpson, Catrine Tudor-Locke, Ashlene Wright, Frank Kee

**Affiliations:** 1 Centre for Public Health, School of Medicine, Dentistry and Biomedical Sciences, Queen’s University Belfast, Belfast, UK; 2 UKCRC Centre of Excellence for Public Health (Northern Ireland), Belfast, UK; 3 AgeNI, Belfast, UK; 4 Health Promotion Research Group, School of Health Sciences, University of East Anglia, Norwich, UK; 5 Later Life Training Ltd, Northumberland, UK; 6 Centre for Health and Rehabilitation Technologies, Institute of Nursing and Health, School of Health Sciences, Ulster University, Newtownabbey, UK; 7 School of Physiotherapy, University of Otago, Dunedin, New Zealand; 8 Community Development and Health Network, Newry, UK; 9 Centre for Physical Activity and Health Research, Ulster University, Newtownabbey, UK; 10 Psychology Research Institute, Ulster University, Coleraine, UK; 11 Department of Kinesiology, School of Public Health and Health Sciences, University of Massachusetts Amherst, Amherst, MA USA

**Keywords:** Randomised controlled trial, Physical activity, Peers, Older adults, Pilot study

## Abstract

**Background:**

Levels of physical activity decline with age. Some of the most disadvantaged individuals in society, such as those from lower socio-economic position, are also the most inactive. Increasing physical activity levels, particularly among those most inactive, is a public health priority. Peer-led physical activity interventions may offer a model to increase physical activity in the older adult population. This study aims to test the feasibility of a peer-led, multicomponent physical activity intervention in socio-economically disadvantaged community dwelling older adults.

**Methods:**

The Medical Research Council framework for developing and evaluating complex interventions will be used to design and test the feasibility of a randomised controlled trial (RCT) of a multicomponent peer-led physical activity intervention. Data will be collected at baseline, immediately after the intervention (12 weeks) and 6 months after baseline measures. The pilot RCT will provide information on recruitment of peer mentors and participants and attrition rates, intervention fidelity, and data on the variability of the primary outcome (minutes of moderate to vigorous physical activity measured with an accelerometer). The pilot trail will also assess the acceptability of the intervention and identify potential resources needed to undertake a definitive study. Data analyses will be descriptive and include an evaluation of eligibility, recruitment, and retention rates. The findings will be used to estimate the sample size required for a definitive trial. A detailed process evaluation using qualitative and quantitative methods will be conducted with a variety of stakeholders to identify areas of success and necessary improvements.

**Discussion:**

This paper describes the protocol for the ‘Walk with Me’ pilot RCT which will provide the information necessary to inform the design and delivery of a fully powered trial should the Walk with Me intervention prove feasible.

**Trial registration:**

*ISRCTN Number* ISRCTN23051918. Date of registration, November 18, 2015.

## Background

Many countries, including the United Kingdom (UK), are facing rapid and sustained increases in the proportion of the population aged 65 years or older [1]. The age-related decline in function, quality of life, and increased risk of morbidity, disability and mortality [2] may be offset or delayed by the adoption of more active lifestyles [3]. A physically active lifestyle in older adults is associated with a reduced risk of developing numerous chronic non-communicable diseases [4, 5] and all-cause mortality [6], and a reduction in the risk of falls [7] and sarcopenia [8]. In addition, regular activity has been linked to improved cognitive function and mental health and wellbeing [9, 10] and higher levels of health-related quality of life [11]. However, despite compelling evidence of the benefits of physical activity as we age, two thirds of the adult population report being inactive, with only 22% of 60–64-year-olds, and as few as 7% aged 75+ years engaging in recommended levels of physical activity in Northern Ireland [12]. Therefore, increasing physical activity levels, particularly among the most inactive, is an important aim of current public health policy in the United Kingdom [13].

Several systematic reviews have summarised the evidence about the effectiveness of physical activity interventions among community-dwelling older adults on self-reported physical activity [14–17]. In general, the interventions encouraged older adult to perform a mode of aerobic activity, of which walking was the predominant form. Most of the interventions included in the reviews were effective at producing short-term changes in physical activity, but levels declined substantially in studies that included longer term follow-up (> 6 months). Individual factors (positive affect and self-efficacy) [18] and social factors (such as social support) [19] are associated with long-term maintenance of changes in physical activity. More recent focus has been on how these factors interact with the potential influence of neighbourhood environments to support physical activity in older adults. A recent study concluded that a supportive physical environment (one which is more ‘walkable’) was associated with higher levels of physical activity, especially in individuals who also had higher self-efficacy and social support [20]. This suggests that an intervention designed on an ecological model, to address multiple levels of influence on physical activity behaviours (including individual, social and environmental factors), is likely to be more effective at delivering sustained changes in activity than interventions targeting individual influences only.

Social cognitive theory (SCT) [21] provides a theoretical framework to understand the relationship between personal, environmental and behavioural factors and has been used effectively to design and implement numerous physical activity interventions. SCT states that personal, environmental and behavioural factors are reciprocally influential in determining behaviour and behaviour change [21]. SCT was used in the design of the Walk with Me intervention. Behaviour Change Techniques (BCTs) identified through a systematic review of peer-led physical activity interventions were mapped onto the core set of psychosocial determinants (i.e. self-efficacy, outcome expectations, goals and impediments and facilitators) of SCT. This approach enabled the research team to design an intervention where the peer mentors’ roles were explicit and could be planned to effectively influence personal, environmental and behavioural factors and their role in physical activity behaviour change. Peer mentors are trained, nonprofessional individuals, who share similar demographic characteristics to the target population (e.g. age, life circumstances and cultural background) and possess experiential knowledge of the target behaviour [22]. In the case of promoting physical activity in the Walk with Me intervention, peer mentors’ roles will be designed to have influence on each of the levels of the socio-ecological model. For example, they may provide individual support and motivation through positive reinforcement and knowledge regarding problem-solving strategies [23].

At present, there are few studies designed to address these multiple influences on the physical activity behaviour of community-dwelling older adults. Therefore, theory-based interventions with multiple components addressing the various levels in the socio-ecological model are required. One approach is to develop multicomponent interventions which capitalise on naturally occurring determinants within target populations and sustainable health promotion mechanisms (i.e. peer groups and their influence on physical activity) to promote long-term physical activity behaviour change. Peer-led interventions offer a model that could help overcome many of the barriers identified above. Peer-led behaviour change interventions are a common and effective means of encouraging behaviour change, including physical activity [24]. Peer mentors will be trained to deliver tailored information about changing physical activity and its benefits. They could provide the necessary social support for behaviour change [25], outside of perceived intimidating settings (e.g. gyms). At an environmental level, the intervention will capitalise on the peer mentor’s knowledge of the local area, by having them engage in activity with the participant in their local neighbourhood, (re)familiarising the participant with local opportunities to engage in physical activity.

### Aims and objectives

The study aims to test the feasibility of a complex peer-led, multicomponent physical activity intervention in socio-economically disadvantaged community-dwelling older adults in a pilot RCT.

The objectives of the study are as follows:

1. To test the recruitment, training and management of a group of peer mentors, working in collaboration with community partners
2. To test the feasibility of a peer-led walking programme targeting inactive older adults in a randomised controlled pilot study, in order to inform the design of a main trial should the trial prove feasible

## Methods/design

The Office for Research Ethics Committees Northern Ireland (ORECNI) has given ethical approval for the study (REC reference number 14/NI/1330).

### Study design and setting

Using the Medical Research Council (MRC) framework for complex interventions [26], we designed a multilevel peer-led physical activity intervention for older adults, tailored to meet the needs of the local community. The intervention package has been developed after defining appropriate BCTs for inclusion in the intervention. They were used to inform the intervention design by (i) identifying BCTs used in previous peer-led interventions. To identify BCTs employed in previous peer-led interventions, a rapid review of the literature was conducted and the use of BCTs was extracted; (ii) conducting interviews with members of the target population to explore their preferences for, and the perceived feasibility of particular BCTs identified from the rapid review. Based on the outcome of these stages, we will then (iii) conduct a pilot randomised controlled trial (RCT). The trial will provide information on recruitment and attrition rates, intervention fidelity, data on the variability in objective physical activity measurements and the resources needed to support the development of a definitive trial [27].

#### Phase 1: identification of approaches used in previous peer-led interventions

The first phase in the complex intervention model was to gather relevant evidence and theory to develop a logic model for the implementation of the intervention, which included the proposed causal pathways and relevant outcome measures. A rapid review [28] approach was used to update a previous systematic review of peer-led physical activity interventions in adults aged over 18 years [24]. The review was not restricted to interventions only targeting older adults as there have been very few peer-led interventions in this age group, and this would limit the inclusion of potentially useful components. We adopted the same search strategy as that used in the previous review [24].

The purpose of the rapid review was to extract the BCTs from the intervention descriptions. BCTs were independently identified from the intervention descriptions by two researchers using the recently published BCT Taxonomy (v1) [29]. Additional details such as intervention setting, target participants, dose, duration, mode of delivery (e.g. individual, group, website, written materials) and country were extracted and used to inform intervention development. The BCTs were mapped onto theoretical domains [30] and to the determinants of physical activity in older adults. They were then used to help identify causal pathways linking interventions to behaviour change using the approach taken in a previous review by Michie et al. [31] and to inform the choice of additional measures (possible mediators of change) for the pilot RCT. The socio-ecological model was used to provide a framework for a multilevel intervention design [32] that addresses multiple levels of determinants including individual, social and environmental factors. In addition to individual factors (such as feedback on current behaviour), we plan to address social factors, by providing peer mentors to act as a social support for change, and environmental factors by matching the programme to local environmental opportunities. The Behaviour Change Wheel [33] was used to map promising BCTs (those that are effective and feasible to deliver within the proposed context) on components of behaviour which reflect these multiple levels: motivation (reflective and automatic), opportunities (physical and social environment) and capability (physical and psychological). The main output at this stage was a shortlist of proposed BCTs to be included in the design of a pilot RCT.

#### Phase 2: feasibility and acceptability of proposed BCTs

In the next stage, we explored the perceived feasibility and preferences for particular BCTs through face to face semi-structured interviews with a purposive sample of 15 older adults from our target communities. This sample include both genders, a range of ages (from 60 to 70 years) and individuals living in different locations and with varying levels of physical activity.

As in a previous study in socio-economically deprived adults [34], participants were presented with a range of hypothetical strategies to promote physical activity. These strategies were presented and explained to participants in interviews, in order to explore their opinion and how these strategies could best be incorporated into the intervention package. Participants were asked to indicate the most and least appealing of strategies. For each strategy, they were asked what they like and do not like about it, whether they think it would result in them being more active and sustaining that activity, what they perceive as potential problems or barriers to its uptake, and where appropriate, how and when the strategy would best be delivered.

In addition to exploring the acceptability of specific evidence-based BCTs, the interviews investigated older people’s experiences of walking, identifying barriers and facilitators which will inform the intervention design [35]. These interviews allowed exploration of views on how the specific behaviour of walking may be promoted in their peer group. Attitudes, beliefs and social perspectives on BCTs may influence engagement with the intervention, and these were therefore explored prior to designing the intervention. Taking account of the interview findings enabled us to avoid or overcome potential barriers within the intervention design and to incorporate elements which are perceived to facilitate walking.

The interviews were conducted on a one-to-one basis, semi-structured and contained between 5 and 8 questions. This semi-structured construct allows participants to focus on specific topics but to express themselves freely in their comments. The number of interviews required was proposed as 15, but data saturation was reached after 12 interviews. Analysis was based on SCT [21], and we used standard thematic analysis with constant comparison methods [36], so that issues arising in the early interviews could be explored in more detail in later interviews. The detailed findings of this stage will be reported separately.

#### Phase 3: pilot RCT

The aim of the trial is to test the feasibility of conducting a trial of a peer-led walking programme in promoting sustained increases in objectively measured physical activity in order to enhance health and mental wellbeing, increase social engagement and improve quality of life in community dwelling older adults.

We will conduct a pilot RCT with 60 inactive, community-dwelling older adults aged 60–70 years, residing in socio-economically disadvantaged communities. In their seventh decade, adults from socio-economically disadvantaged areas often transition from good health to poor health, from being fit to being unfit, from independence to dependence and may transition from employment to retirement [37]. This age range (60–70 years) therefore can be seen as a transition period, as older adults from socio-economically disadvantaged areas transition from good health to poor health.

Physical activity interventions delivered at points of life transition such as changes in social (loss of companions), economic (retirement) or health circumstances in older adults may be advantageous. Li et al. [37] acknowledged that this ‘transition’ is not necessarily an abrupt change, but may involve a gradual change over time. Whilst retirement from paid employment may lead to change in employment and income, these transitions may not be applicable to many people in disadvantaged communities. Thus, in addressing the identified research priorities of inequalities in health and physical activity participation in socio-economically disadvantaged communities, we will include all 60–70-year-olds in such communities in our target population rather than focusing on retirement.

For this study, socio-economically disadvantaged communities are defined as those falling within the lowest quartile of super output areas (geographical areas of consistent size used to facilitate the calculation on deprivation indices), based on the Northern Ireland Multiple Deprivation Measure (NIMDM) (https://www.nisra.gov.uk/). For ease of administration, the pilot study will be conducted in the South-Eastern and Northern Health & Social Care Trusts, which covers a large geographical area and a mix of urban and rural settings. In doing so, the intervention would fill an identified gap in preventive service provision for older adults who may need support to increase their physical activity levels in order to maintain physical function and independence in daily living.

### Participant recruitment

**Fig. 1:**
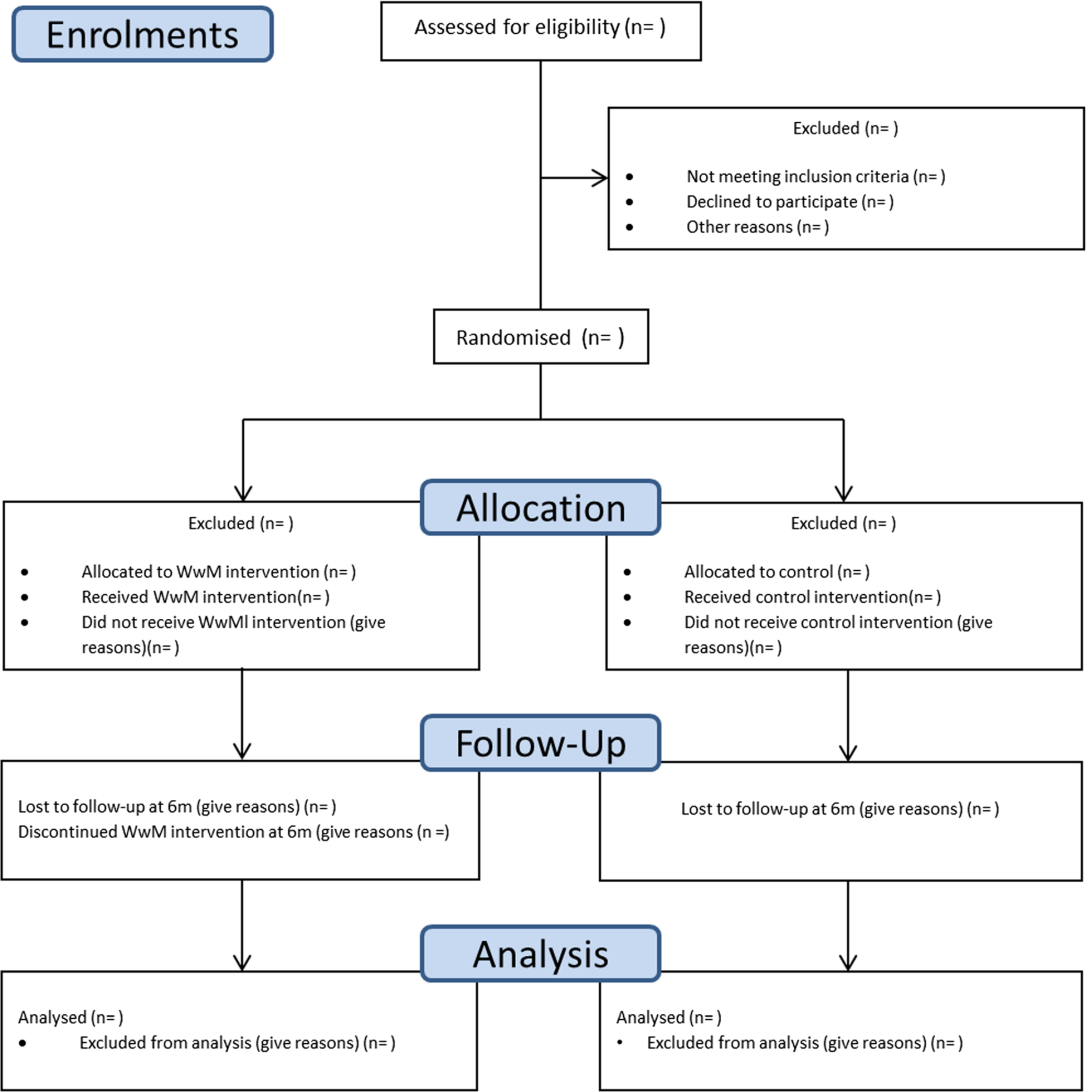
CONSORT flow diagram for the Walk with Me trial

We aim to recruit 60 participants. Previous research has identified difficulties in recruiting participants from socio-economically disadvantaged communities [38], and thus, a wide range of active and passive recruitment strategies are required. Active strategies will include identification and referral of potential participants through project partners. Passive recruitment methods will include sending study information, along with a letter from their General Practitioner to suitable patients from primary care practices in target communities, distribution of leaflets and posters through primary care practices, community centres, libraries, health centres, faith-based groups and churches, and the email lists and social media outlets of project partners. Those eventually recruited will be asked how they learned of the study. Individuals who wish to participate will be asked to contact the study team by telephone, in writing or by email. The flow of participants through the trial is described in Fig. 1.

#### Inclusion criteria

Participants will be eligible for the pilot trial if they meet the following inclusion criteria:

- Male or female aged 60–70 years
- Living in a socio-economically disadvantaged community (defined as the lowest quartile of super output areas according to the NIMDM)
- Competent to give informed consent
- Not currently physically active (assessed using the General Practice Physical Activity Questionnaire [39])
- Community-dwelling (i.e. living in their own home)
- Planning to stay in the current residence during the next year
- Able to communicate in English.
- No self-reported recent history of myocardial infarction, stroke or physical limitations that would limit ability to participate in a walking programme (assessed using the Physical Activity Readiness Questionnaire [40]).

#### Allocation and randomisation

After contacting the study team, potential participants’ meet with a member of the project team to discuss the study in detail. At this face to face contact, all eligible participants are informed about the details of the project, including what the intervention and the control group will receive and what outcomes are collected. They are advised that participation in the project is voluntary and that they have the right to withdraw consent, at any time, without the need for explanation. They are given a minimum of 48 h to consider participation in the study. After this, those who wish to take part are asked to read, complete and sign a consent form, which will be returned to, and countersigned by a member of the research team. Eligible participants complete baseline outcome measures and are then randomised to an intervention or control group (1:1 ratio) using block randomisation with randomly permuted block sizes. The Northern Ireland Clinical Trials Unit (NICTU) trial statistician will generate the randomisation sequence which will be concealed in sealed envelopes opened by a member of the research team responsible for pairing the mentors and intervention group participants, after completion of baseline measures.

### Walk with Me intervention

The 12-week intervention begins with a face-to-face meeting between the peer mentor, the participant and a member of the research team whose role is to facilitate initial discussions. At this introductory meeting, there is a discussion of the peer mentor’s role and the main behaviour change components of the intervention are described (goal setting, reviewing and setting behavioural goals, problem-solving). The participant is given a pedometer to keep (Yamax SW-200, Yamax Corp, Japan) and a participant information and resource booklet which contains study contact details, weekly step diaries and a physical activity action planning template. The purpose of the pedometer is to allow the participant to set weekly step goals and monitor their own progress in achieving these. The participant familiarises themselves with the function of the pedometer and completes a small walk with the peer mentor to validate the accuracy of the device and ensure they understand how to use it. The meeting concludes with an exchange of contact details and plans to meet the following week.

The initial period of the intervention (activation stage, weeks 1–4) is designed to enable the participant and peer mentor to establish a rapport (e.g. by building a trusting relationship that is necessary for successful peer mentoring).

During this period, the participant will record their baseline levels of physical activity, set early short-term goals and action plan to incorporate additional physical activity into their weekly schedule by agreeing to meet to walk with their mentor (at a minimum of once every two weeks for 45 mins-1 h) and discuss physical activity goals/goal setting and problems/barriers to increasing physical activity with their mentor (weekly face-to-face or over the phone). This period enables the peer mentor to advise the participant of the frequency, intensity, time and type of physical activity they should be taking part in (e.g. by discussing the physical activity guidelines for older adults (copies of which are included in the participant information and resource booklet).

The programme continues (behavioural practice stage, weeks 5–8) with the participant and mentor meeting at least once every 2 weeks to walk and discuss goals/barriers to increasing physical activity. This meeting takes place at a venue of the mentor and participants choosing. This enables the mentor to demonstrate the appropriate walking pace to achieve moderate intensity physical activity and enables the participant to set individual physical activity goals by taking into consideration their capabilities. For the purposes of this study, moderate intensity activity is defined as walking at a pace that leaves the participant slightly breathless but still able to hold a conversation. Weekly activity targets are reviewed and agreed. If the participant is having difficulty increasing their physical activity, the mentor will discuss strategies to overcome barriers to increasing physical activity (e.g. by discussing opportunities for physical activity in the local neighbourhood environment). During this period the mentor and participant begin to plan a walk with a local community based/leisure centre-based walking group (to take place during weeks 10–12) that will facilitate the participant to continue to increase/maintain their activity level when the structured component of the intervention comes to an end.

The final 4 weeks (habit formation stage, weeks 9–12) of the intervention emphasise behaviour rehearsal and practise by walking regularly in a locally accessible physical activity environment (e.g. local park). In order to increase physical activity habit formation, the peer mentor prompts rehearsal and repetition of physical activity behaviour by meeting with/discussing physical activity goals with the participant (e.g. via weekly/biweekly walks/telephone contacts). The final weeks of the structured component of the intervention provide an opportunity for the participant and mentor to discuss other community-based physical activity opportunities and to attend a local community group (e.g. men’s shed) to facilitate the maintenance of physical activity behaviours at the conclusion of the 12-week intervention.

### Peer mentor training

Peer mentors will be recruited concurrently to participant recruitment through partnerships with local community organisations and charitable organisations. Before being appointed as a peer mentor, these individuals will attend a meeting with a member of the research team at a local community venue where they will be provided with information on the study and their role within it. During this session, potential peer mentors will be asked about their willingness to undergo the required training to deliver the programme and their attitude and commitment to helping others increase their physical activity levels. This session will give the mentor an opportunity to discuss their interests, which in turn helps with the process of pairing the peer mentor with a potential participant. Peer mentors will complete approved background checks prior to being matched with potential participants.

Peer mentors will receive training (2 × 1 h standardised training sessions with the project manager using a peer mentor training and support manual) to develop their skill, knowledge and confidence to promote physical activity among their peers. Training is planned to continue during the programme at regular monthly meetings with mentors, such as providing guidance and support during mentoring sessions. During the training sessions, mentors will receive information on important programme responsibilities. These included a peer mentor’s commitment, main tasks and requirements; information about physical activity guidelines for older adults; education about BCTs and their role in the programme; modelling physical activity behaviours; helping their peer complete programme activities; and reporting on activities or providing feedback to the project team. Mentors will be trained on how to build and sustain an effective mentoring relationship with a peer, as well as skills building in the areas of active listening, communication and providing social and emotional support. A full list of the BCTs to be delivered in the intervention is detailed in Table 1.

**Table 1:** BCTs in the Walk with me Intervention

Grouping and BCTs (expanded)	Intervention activities
Goals and planning	
Goal setting (behaviour)	Peer mentors will set or agree on a goal defined in terms of the behaviour to be achieved
Action planning	Peer mentors will prompt detailed planning of performance of the behaviour by including specific reference to include (at least one of) context, frequency, duration and intensity of physical activity. Context may be environmental (physical or social) or internal (physical, emotional or cognitive)
Problem-solving	Peer mentors will analyse or prompt the person to analyse factors influencing the behaviour and generate or select strategies that include overcoming barriers and/or increasing facilitators
Review behaviour goals	The peer mentor will review behaviour goal(s) jointly with the person and consider modifying goal(s) or behaviour change strategy in light of achievement
Behavioural contract	Peer mentors will create a written specification of the behaviour to be performed, agreed on by the person and witnessed by another
Feedback and monitoring	
Self-monitoring of behaviour	Peer mentors will distribute pedometers and step diaries to the people that they are mentoring so that they may monitor and record their physical activity behaviour(s) as part of the intervention
Social support	
Social support (practical)	Peer mentors will advise on, arrange or provide practical help for the performance of the behaviour
Social support (emotional)	Peer mentors will advise on, arrange or provide emotional social support for the performance of the behaviour
Shaping knowledge	
Instruction on how to perform the behaviour	Peer mentors will advise or agree on how to perform the behaviour
Natural consequences	
Information about health consequences	The peer mentor will provide information (e.g. written, verbal, visual) about health, consequences of performing the behaviour
Information about social and environmental consequences	The peer mentor will provide information (e.g. written, verbal, visual) about social and environmental consequences of performing the behaviour
Comparison of behaviour	
Demonstration of the behaviour	Peer mentors will provide an observable sample of the performance of the behaviour
Repetition and substitution	
Behavioural rehearsal and practice	Peer mentors will prompt practice or rehearsal of the performance of the behaviour one or more times in a context or at a time when the performance may not be necessary, in order to increase habit and skill
Habit formation	Peer mentors will prompt rehearsal and repetition of the behaviour in the same context repeatedly so that the context elicits the behaviour
Graded tasks	Peer mentors will set easy-to-perform tasks, making them increasingly difficult, but achievable, until behaviour is performed
Antecedents	
Adding objects to the environment	The provision of pedometers will add objects to the environment in order to facilitate performance of the behaviour
Restructuring of the social environment	Peer mentors will change or advise to change the social environment in order to facilitate performance of the behaviour

Peer mentors will also receive a Public Health Agency Information Leaflet ‘Ageing well by being active everyday’ (http://www.publichealth.hscni.net/publications/ageing-well-being-active-every-day) which contains brief information on the physical activity guidelines for older adults and brief advice on how to become more active. Before delivering the programme, outcome measures similar to those collected from participants are recorded including age, sex, physical activity measured with an Actigraph GT3X+ accelerometer, self-rated physical and mental health, social engagement, loneliness, and physical and social activity self-efficacy and outcome expectancy (see below for details).

### Control group

Those assigned to the control group will not receive any additional support to change their activity over the course of the intervention period. They will receive a Public Health Agency Information Leaflet ‘Ageing well by being active everyday’ (the same booklet that will be given to the intervention group). Participants in this group will be informed that after the 6-month data collection point, they will not be offered the opportunity to participate in the intervention; however, they will be given the opportunity to discuss with a member of the project team other community-based physical activity opportunities to facilitate them to change their physical activity behaviour (e.g. information and advice on activity/walking groups that regularly met in that individuals local community/leisure centre).

### Outcome measures

Outcome measures will be assessed at baseline (prior to randomisation), after completing the intervention (12 weeks) and 6 months after baseline (Table 2). All outcomes have been chosen in order to inform the development of a future definitive trial. We plan to assess the feasibility and acceptability of employing these measures.

**Table 2:** Outcome measures and time points in the Walk with Me trial

Outcome	Measurement tool	Time points
MVPA	GT3X+ accelerometer	Baseline, 12 weeks, 6 months
Self-reported physical activity	EPAQ-2	Baseline, 12 weeks, 6 months
Physical and mental health	SF-12	Baseline, 12 weeks, 6 months
Mental wellbeing	Warwick-Edinburgh Mental Well-being Scale	Baseline, 12 weeks, 6 months
Health-related quality of life	EuroQol-5D	Baseline, 12 weeks, 6 months
Loneliness	UCLA Loneliness Scale	Baseline, 12 weeks, 6 months
Social engagement	Lubben Social Network Scale	Baseline, 12 weeks, 6 months
Health and social care services resource use	Annotated Cost Questionnaire	Baseline, 12 weeks, 6 months
Acceptability of the intervention	Post-intervention exit questionnaire	6 months
Self-efficacy	Physical Activity Self-Efficacy Measure [49] Social Activity Self-Efficacy Measure (adapted from [49])	Baseline, 12 weeks, 6 months
Outcome expectancies	Physical Activity Outcome Expectancy Measure (Items 1–12 from [50]) Social Activity Outcome Expectancy Measure (Adapted from [50])	Baseline, 12 weeks, 6 months

The primary outcome measure will be mean daily minutes of moderate and vigorous physical activity (MVPA) objectively measured over 7 days using a waist-worn Actigraph GT3X+ accelerometer. Participants will be asked to wear the accelerometer on an elasticated belt, during waking hours, for seven consecutive days. To explore the context of changes in physical activity and the types of physical activity that participants report performing, a validated self-reported physical activity questionnaire (EPAQ-2) [41] will be self-completed.

**Fig. 2:**
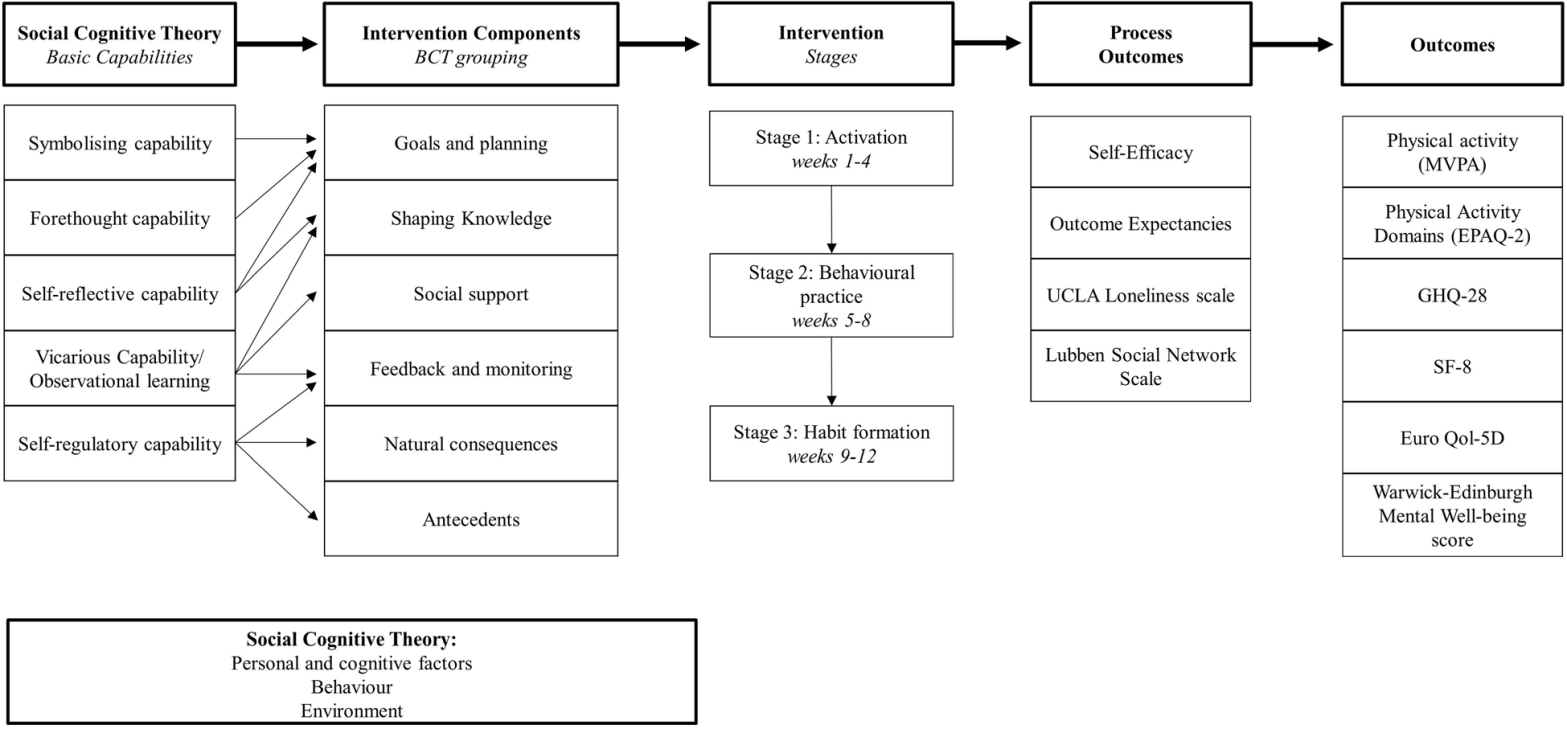
Walk with Me study logic model

Informed by the intervention logic model (Fig. 2), secondary outcomes will include physical and mental health and mental wellbeing using the Short-Form 12 (SF-12) Health Questionnaire [42], the 28-item General Health Questionnaire (GHQ-28) [43] and the Warwick-Edinburgh Mental Well-being Scale [44], respectively. Health-related quality of life will be assessed using EuroQol-5D [45]. Social engagement will be measured with the UCLA Loneliness Scale [46] and the Lubben Social Network Scale [47]. Physical activity and social activity self-efficacy [48], and physical activity and social activity outcome expectancies will also be measured [49]).

Although the trial is not designed to estimate cost-effectiveness, we will pilot the use of participant health and social care services resource use instruments. These will inform the design of the economic evaluation of any subsequent RCT, developed using items from the Annotated Cost Questionnaire [50]. The health and social care services resource use instrument will be given to participants at the outset of the trial, and they will be asked to return it at the 6-month assessment, recording their use of services over that period.

### Process evaluation

In line with MRC guidance on process evaluation [51], we will assess whether the intervention was delivered and received as intended (implementation), how intervention activities, and participants’ interactions with them impacted behaviour change (mechanisms of impact; through behavioural processes as per logic model––Fig. 2); and how potential external factors may have influenced the delivery and functioning of the intervention (context; through post-intervention focus groups).

Process evaluation will assess whether the intervention was delivered and received as intended. This will be achieved through structured observation of intervention delivery (by a member of the research team responsible for mentor training), semi-structured interviews and focus groups with peer mentors and participants, and review of intervention records and participant diaries.

Intervention implementation fidelity will be assessed in the following ways. For each peer mentor, one randomly selected first meeting and follow-up meeting will be audio-recorded and assessed by the research team for content and delivery fidelity, using a quality assurance form.

Feedback on fidelity will be given to each peer mentor during the intervention to assist them in delivery. Completeness in the dose of delivery of the intervention will be assessed by asking the peer mentors and a sample of 10 trial participants to complete weekly checklists and record a diary of contacts (both face-to-face and telephone). The diary will include information on the number of attempts to make contact and the duration of each successful contact. This information will be summarised at the end of the intervention in line with the studies fidelity assessment and implementation plan [52] to assess overall fidelity.

To assess theoretical mediators of physical activity behaviour (mechanisms of impact), physical activity and social activity self-efficacy and physical activity and social activity outcome expectancies will be measured (see study logic model––Fig. 2).

Post-intervention focus groups and semi-structured interviews will be used to provide context to the research by examining how potential external factors may have influenced the delivery and functioning of the intervention.

### Feasibility and acceptability of the intervention

The acceptability of the intervention will be assessed using two approaches. Firstly, all participants will be asked to complete a post-study exit questionnaire, as used in a previous physical activity intervention [53]. This questionnaire asks participants to rate their experience of the intervention and provide reasons for their decision to take part. Other items assess participant satisfaction with the advice/information they received about this study (including the participant information sheet and other information). If the majority of responses are positive, we would assume that the intervention is acceptable; otherwise, we would reflect on how the intervention may be better optimised.

All participants in the intervention group will be invited to attend focus groups or one-to-one semi-structured interviews with an independently appointed researcher to discuss their views on the feasibility and acceptability of the intervention. This will allow us to explore acceptability in greater depth than may have been captured on the exit questionnaire. Focus group/interview participants will be asked to explore reasons for success and challenges to increasing their physical activity and what they would change about the intervention if they were to take part in it again. Participants will not be offered compensation for taking part of the study.

Peer mentors will be invited to attend separate focus groups or one-to-one semi-structured interviews to provide feedback on their experiences of the intervention. Topics will include challenges to intervention delivery, perceived success, and barriers to implementation and suggestions on how to improve the intervention. Primary questions will relate to the different BCTs employed, reviewing each in turn, considering what worked to increase engagement in walking for some individuals and what did not work for others. Control group participants will be invited to attend semi-structured interviews where they can give their feedback on their involvement and their motivation to take part in the research.

Data from the focus groups and interviews will be transcribed verbatim and assessed using content and thematic analysis [36]. These focus groups and interviews will further inform the development and design of a fully powered trial by enabling appropriate refinement of the intervention’s components and delivery for the subsequent RCT, if deemed appropriate. This feedback will also provide an in-depth examination of barriers to and compliance with the implementation of the protocol. The qualitative data thus obtained will help to explain quantitative data regarding recruitment and retention, collected during the process of the intervention, and responses written in the exit satisfaction questionnaire.

The feasibility of conducting a definitive trial, defined as the ability to recruit participants within the time frame, and retain a significant proportion of them within the trial, will be assessed based on the recruitment and attrition rates and the qualitative feedback from participants and mentors. The recruitment rate will be calculated by totalling the number of participants recruited as a proportion of the pre-defined target of 60 participants, within the timeframe of the study. Attrition will be measured as the proportion that does not complete outcome measures at 6 months after baseline (either because they dropped out or failed to complete outcome measures).

#### Assessment of harms

This is a low risk intervention, and we do not anticipate any serious adverse events. However, participants will be encouraged to report adverse events resulting from activity (e.g. musculoskeletal problems, shortness of breath or falls) on an ongoing basis by contacting the study team. Adverse events reported by participants will be recorded on a standard proforma [54].

### Data analyses

As this is a pilot study, significance tests of change over time will not be performed on the primary of secondary outcomes. Intervention effects will be represented by point estimates, and both 85 and 95% confidence intervals will be estimated at each follow-up time point [55]. Analysis will be conducted by a researcher blind to group allocations. Recruitment, retention and adherence rates will be reported and any adverse events recorded and, alongside effect size, will be used to estimate a sample size required for a definitive trial, if deemed appropriate. All semi-structured interviews and focus groups will be audio-recorded and transcribed verbatim for further analysis. Transcripts from audio recordings will be analysed using standard thematic analysis techniques [36]. At least two qualitative researchers will structure the data and subsequent interpretation. Themes will be compared to the results of the post-study exit questionnaires to make a judgement about the overall acceptability of the intervention.

## Discussion

This study focuses on the need to address declining levels of physical activity in the older adult population. The Walk with Me trial aims to test the feasibility of a theory-based peer-led intervention to increase the physical activity of inactive community-dwelling older adults from areas of socio-economic deprivation. The study design and evaluation was informed by the MRC framework for complex interventions, and is based on extensive prior development work, including a systematic review and semi-structured interviews with members of the target population. In addition, the emphasis is on rigorous evaluation using objective and self-reported outcome and process measures. The data collected in this pilot trial will inform the design and delivery of a fully powered definitive trial if deemed appropriate.

## Abbreviations

BCT: Behaviour Change Technique
GHQ: General Health Questionnaire
MRC: Medical Research Council
MVPA: Moderate to Vigorous Physical Activity
NICTU: Northern Ireland Clinical Trials Unit
NIHR: National Institute of Health Research
NIMDM: Northern Ireland Multiple Deprivation Measure
RCT: Randomised controlled trial
SCT: Social cognitive theory
SF-12: Short-Form 12
